# Lavage of the Intestinal Tube

**Published:** 1893-12-16

**Authors:** 


### LAVAGE OF THE INTESTINAL TUBE.

The clinical aspects of intestinal antiseptics have been recently discussed in these columns, but the difficulties in arriving at complete asepsis are found to be so great that any method which promises to help us in arriving at a perfect procedure must receive our careful consideration. A method by which the whole of the digestive tract may be irrigated has been worked out and practised by Lesage and Dauriac. It enables us to treat a certain class of cases with great rapidity and certainty, and would appear to have considerable value. Von Genersich has simultaneously used a somewhat similar plan.*

It was discovered that a stream of water could be sent from the rectum into the stomach, and then by an œsophageal tube if desired carried out through the mouth, provided that the patient were kept horizontal with the left hip raised, and the stream maintained under a regular and weak pressure of from 20 to 50 centimetres (8 to 20 inches). After trials had been made of this method on the dead body and on animals and its practical value and easiness demonstrated fully, eleven children who were suffering from an intractable fœtid diarrhœa were treated by complete lavage of the intestinal tract in the same way. A weak solution of lactic acid was thus injected, and either allowed to flow back or siphoned off through the mouth. In all cases the diarrhœa stopped after the first lavage, and if any relapse occurred a single washing was sufficient to complete the cure. Similar good results were obtained by creoline solutions. Five new-born children suffering from *diarrhée verte* were cured by a single irrigation with a lactic acid solution. Two adults affected with catarrhal jaundice were treated by complete lavage, and on the next day the clay coloured stools were replaced by those containing bile; an entire recovery followed. A typhoid case received regular daily modified injections (not exceeding five litres each) of warm water under a very moderate pressure. The tongue cleaned at once, headache disappeared, and with a low temperature the patient made a rapid recovery. As Messieurs Lesage and Dauriac point out, the method might be of great use before surgical operations in rendering the whole of the digestive tube aseptic; or again, in some forms of intestinal obstruction, or when we wish to obtain a thorough purgation. It is especially valuable in uræmia and states of auto-intoxication, for nutrient enemata, and in cholera. Drs. Bousey and Von Genersich have used a similar plan in cholera in several cases, but the latter employs a pressure of from 80 to 100 centimetres, which Lesage and Dauriac object to. Under their method the intestines are not distended or even filled with fluid. Air remains in the upper parts of the coils. Percussion shows a cushion of air in the umbilical region, which has a protective value while aiding in the flow of the fluid. The heart and lungs, they say, do not suffer any pressure, since there is no abdominal distension, and by experiments on the dead body, they found that the diaphragm is not pushed up, nor does the fluid enter the biliary canals.

It is well known that Senn injects hydrogen gas per rectum as a means of discovering any wound of the intestines, and that this passes out through the mouth if they are intact. Cantani, too ("Practitioner," 1880), gave an account of two instances where injections of oil entered the stomach and were vomited, so that the possibility of the proceeding is not to be denied. Professor Struthers was accustomed to account for cases like those of Cantani by supposing that the iliocæcal valve was defective, and referred to the fact that air from a distended colon could not be forced back through the valve in the dead body. It is this absence of distension, combined with a tilted position—the left hip being elevated—that Lesage and Dauriac insist on as essential in their injections. Though they advise it, a very long enema tube is not needed, for, as Treves and others have shown, no tube can be passed, as a rule, beyond the sigmoid flexure. Messieurs Lesage and Dauriac direct that a vessel containing some six or eight litres, with a soft siphon tube carried into the rectum, be placed so that its surface is not more than eight to twelve inches higher than the body of the patient. The liquid is allowed to run quietly, and under this very small pressure soon fills the cæcum as well as the transverse colon. The filling of the cæcum goes on gradually, but without causing any distension. Palpation and percussion show only moderate fulness. This obtained, the fluid mounts, they consider, to the small intestine when the third litre is beginning to be injected. About this time the patient feels some intestinal colic, and the slight pain felt shows that the small intestine is reached. When three litres have entered, the person in charge of the flask watches the surface of the water. If it ceases to fall he elevates the vessel very slightly, and gauges the pressure by following carefully the surface with his eye, raising it a little or depressing as necessary. The entrance of the water is not as simple as might be imagined. It is needful to note the important part played by flatus in the intestine. In each fold of gut the fluid occupies the lower and the gas the upper part. Following indications given by the surface of the water, we await the gradual spontaneous spread of the fluid through the entire small intestine. When this is full, dulness appears on the right side and above the bladder next over the side of the abdomen. On the other hand, around the umbilicus, the abdomen becomes prominent and resonant. This is due to the collection of gas, which forms an aerial cushion around the umbilicus. In consequence of the horizontal position of the patient, the fluid runs through the whole intestine, and that without distending it until, at the beginning of the sixth litre, it penetrates into the stomach. The patient immediately then suffers from nausea, and vomits the injected fluid soiled by fæcal matters. This is the best sign of the entry of the liquid into the stomach. Traube's space may become dull. It is of the greatest importance to note the surface of the fluid as an index of the pressure, and to allow the

* *Lancet*, October, 1893.

intestine, so to speak, to draw in the fluid, raising the vessel to even 40 or 50 centimetres. After the fluid has reached the stomach to the amount of one or two litres, the sound can be withdrawn, and at once a flood of liquid escapes by the anus. We may instead permit it to flow out through the sound by depressing the bowl, and then repeat the lavage with a fresh supply; or we may pass an œsophageal sound, so that the fluid runs out as through a siphon, passing the whole distance from the rectum through the mouth. The important points are the use of a large quantity of fluid; the weak pressure employed, so that the fluid spreads itself along the intestinal tract spontaneously; the slowness of the stream, the horizontal position of the patient, and the depressed position of the cæcum. It is only necessary to add tannin, lactic acid, or benzo-naphthol to render the intestines aseptic.

The amount of fluid used has varied with the age of the patient. About one litre was employed for new-born children, two litres for those a little older, and eight litres for adults. The guide is the appearance of vomiting, and the variations in the surface of the water.

The view is that a high tension of air and water closes the iliocæcal valve, and that it is under conditions of high tension that its functions have been studied, and that thus a greater activity has been attributed to it than it normally possesses. The position of the patient is of high importance, since our chief aim must be to avoid causing a high pressure of gas by the injected fluid. The tilted position of the patient facilitates the escape of gas into the transverse colon, the valve opens as soon as the tension ceases, and the water passes quietly through the cæcum into the small intestine.

Whether this view of the action of the valve be correct or not, the number of cases in which these experimenters and Von Genersich have injected fluids into the stomach, both in the dead body and in the living, shows that the difficulty arising from the valve is not insurmountable, and their method may be of great use in the cases indicated. It should never be attempted under chloroform, as far as present experience goes, and if a patient complains much of colicky pains during the injection they advise us to stop and wait the cessation of the disturbance. In one patient to whom complete lavage was being administered an entire tapeworm came away.

The anus may be compressed during the injection, or Lund's air pad may be employed, or a similar contrivance invented by Messieurs Lesage and Dauriac, for use in giving these injections. It is hardly needful to remark upon the danger of the proceeding when any ulceration of the bowel is present, but with reasonable care there does not seem any great risk of over distension in a healthy bowel.

Now the methods of Lesage and Dauriac present many obvious disadvantages, and these are of such a nature that it is hardly likely that their practice will be widely adopted and certainly only in desperate cases. The great point to notice is the proof that the entire intestinal tract can be irrigated *per anum.* Now there are many affections in which the disease is situated in the lower half of the small intestines and in the upper part of the larger. A modification of their method, viz., the introduction of sufficient fluid to reach the seat of the lesion instead of reaching as high as the stomach, offers so many advantages that we may confidently advocate the procedure in certain cases, such as enteric fever and tubercular disease of the intestine.

The advantages of irrigation of the colon in infants have been clinically investigated by Huddleston*, of New York, and his results have a special interest in connection with irrigation of the entire intestinal tract. The child lies on the mother's lap, face down, with its legs hanging by her side. A two-quart fountain syringe filled with a salt solution of nearly normal strength (six per cent.) is hung about four feet above the baby, the water being at the temperature of 68 to 75 degrees F. as it flows from the funcet. A nozzle anointed with vaseline is passed up the rectum a considerable distance, and the water allowed to flow so as to fill the colon. The amount of water retained varies from a few ounces to two quarts. When a quart or more is retained the child generally vomits the contents of the stomach, but in no case has it been evident that the water reached the stomach through the intestine. After no less than fifty cases of diarrhœa in children under two years of age had been treated in this manner, none of them picked cases, it was found that sixty-six per cent. were relieved by one irrigation, some were washed out twice, and a few three times.

Now these cases illustrate most forcibly our contention that the method of irrigation merits our fuller consideration, and that we may hope for much practical advantage from a modification of the method for lavage of the entire tract.

* *New York Med. Rec.*, Sept. 9, 1893.

